# Sexual Recruitment in *Zostera marina*: Progress toward a Predictive Model

**DOI:** 10.1371/journal.pone.0138206

**Published:** 2015-09-14

**Authors:** Bradley T. Furman, Bradley J. Peterson

**Affiliations:** School of Marine and Atmospheric Sciences, Stony Brook University, Stony Brook, New York, United States of America; University of California Santa Cruz, UNITED STATES

## Abstract

Ecophysiological stress and physical disturbance are capable of structuring meadows through a combination of direct biomass removal and recruitment limitation; however, predicting these effects at landscape scales has rarely been successful. To model environmental influence on sexual recruitment in perennial *Zostera marina*, we selected a sub-tidal, light-replete study site with seasonal extremes in temperature and wave energy. During an 8-year observation period, areal coverage increased from 4.8 to 42.7%. Gains were stepwise in pattern, attributable to annual recruitment of patches followed by centrifugal growth and coalescence. Recruitment varied from 13 to 4,894 patches per year. Using a multiple linear regression approach, we examined the association between patch appearance and relative wave energy, atmospheric condition and water temperature. Two models were developed, one appropriate for the dispersal of naked seeds, and another for rafted flowers. Results indicated that both modes of sexual recruitment varied as functions of wind, temperature, rainfall and wave energy, with a regime shift in wind-wave energy corresponding to periods of rapid colonization within our site. Temporal correlations between sexual recruitment and time-lagged climatic summaries highlighted floral induction, seed bank and small patch development as periods of vulnerability. Given global losses in seagrass coverage, regions of recovery and re-colonization will become increasingly important. Lacking landscape-scale process models for seagrass recruitment, temporally explicit statistical approaches presented here could be used to forecast colonization trajectories and to provide managers with real-time estimates of future meadow performance; i.e., when to expect a good year in terms of seagrass expansion. To facilitate use as forecasting tools, we did not use statistical composites or normalized variables as our predictors. This study, therefore, represents a first step toward linking remotely acquired environmental data to sexual recruitment, an important measure of seagrass performance that translates directly into landscape-scale coverage change.

## Introduction

Seagrass landscapes are spatiotemporally dynamic [1–3]. Metrics of floral composition [4], shoot density [5], areal coverage [6] and landscape configuration [3] have all been shown to vary widely over seasonal to inter-annual times-scales. Unfortunately, as in other ecologies, natural rates and patterns of variability have been eclipsed by modern-era declines [7]. An alarming component of estuarine urbanization, the loss of seagrasses has spurred considerable effort to document and understand changing coverage patterns [8, 9]. Initial work focused on the intuitive relationship between meadow contraction and water quality, and the resultant bio-optical and secchi-depth models explain a large proportion of the spatiotemporal variance [10–12]. Although paradigmatically instructive, the univariate role of light limitation on deep edge of occurrence is, however, insufficient to predict distributions for specific landscapes over annual timescales; that is, while true for regions or time-series spanning large ranges in depth or light penetration, light limitation models are insensitive to typical gradients in light stress [13]. Aimed at more precise forecasting, and fueled by the growing availability of GIS data, contemporary investigators have employed a host of sophisticated numerical and statistical approaches to describe seagrass coverages, viz., habitat suitability [9, 14] and species distribution models [15–18], ecological process models [19–21], and vegetative growth models [22–25]. Together, these efforts have broadened the range of predictor variables (e.g., salinity, slope, wave energy, current flow, tidal range, sediment characteristics, temperature, etc.) and offer potential advantages over traditional mapping techniques [17, 26, 27].

The modeling of seagrass landscapes is often less expensive than physical surveys [17]. Statistical and process models are less subject to the artifacts and limitations of photo-interpretation, and tend to integrate longer time-scales–blurring some of the spatial dynamism confounding static seagrass maps [15, 20]. Models also allow for the evaluation of change, and the assessment of alternate scenarios of climate and human impact [27]. However, nearly all of these approaches implicitly assume that seagrass distributions remain in equilibrium with the local environment [13, 26]. There are a number of reasons why this may not be the case.

First, although seagrass loss can be quite fast (physical disturbances are instantaneous and carbohydrate reserves last mere weeks), the mechanisms of spatial expansion operate on much slower time-scales [28, 29], but see [30]. Viewed largely as a vegetative process, patch growth has been found to match rhizome elongation rates at centimeters to meters per year with strong seasonal cycles in production [21, 31, 32]. Therefore, at landscape scales, environmental forcing of seagrass coverage may not be apparent for some time. A number of investigators have addressed these offsets by exploring time-lagged predictor variables [13, 15, 33]; however, no evidence for systematic delays has yet been found [13], and up to a decade may in fact be necessary for adequate meadow response [26]. Second, we know very little about the mechanisms and relative importance of sexual recruitment in seagrasses [19, 34]. Recent genetic surveys of *Zostera marina* have begun to highlight the role of pollen and seed dispersal in meadow development [35–37]; however, very few quantitative estimates of contemporary gene flow exist, and dispersal kernels for seeds, rafted flowers and vegetative fragments remain poorly constrained for any seagrass species [38, 39]. Nowhere is this more evident than for regions undergoing recovery [6, 40, 41], where the pace and shape of re-colonization can only be described as enigmatic [32]. Without a quantitative understanding of recruitment dynamics, errors of commission will continue to undermine the development and interpretation of spatially explicit distribution models, and to force anecdotal explanations of recovery.

Toward filling this gap, we sought to relate time-lagged climatic variables to recruitment success within a sub-tidal, colonization phase, *Z*. *marina* meadow in Shinnecock Bay, New York, USA. A previous study of the same location revealed that sexual recruitment was much more important than vegetative growth with respect to space acquisition over a 13-year period (2001–2014), and that the rate of recruitment varied widely by year [42]. Concurrent estimates of floral densities and seed production made within the meadow over 3 years (2012–2014) indicated that no substantive changes in reproductive effort or mating system effectiveness had occurred (L. Jackson, unpubl.). Because the system was (1) uniformly shallow and therefore light-replete, (2) unaffected by drift or epiphytic algal growth, (3) mono-specific and binary in composition–i.e., *Z*. *marina* embedded within a sandy matrix, (4) wave exposed and (5) subject to temperature fluctuation, we hypothesized that autecological stress would control seedling survival, and that variability in the physical environment would lead to inter-annual patterns in sexual recruitment.

Using a multiple linear regression (MLR) approach, we examined the association between isolated patch emergence (i.e., seed-borne recruitment) and estimates of relative wave energy, atmospheric condition and water temperature over an 8-year period (2007–2014). Two models were developed, one appropriate for the dispersal of naked seeds, and another for seeds delivered by rafted flowers. To facilitate their use as forecasting tools, we did not use statistical composites (i.e., principal components) or normalized variables (i.e., Z-scores) as our predictors. This study therefore represents a first step toward providing managers with a baseline forecasting tool linking easily acquired environmental data to sexual recruitment, an important measure of seagrass performance that translates directly into landscape-scale coverage change. Although conducted on a relatively small spatial scale (56,250 m^2^), this study applies directly to similar locations within Shinnecock Bay (i.e., sandy, wave-exposed shoals) and stands as a proof of concept, adaptable to other landscapes wherever sufficient seagrass coverage and environmental data exist.

## Materials and Methods

### Study Site

All fieldwork was conducted in Shinnecock Bay, a backbarrier lagoon system in southeastern Long Island, New York, USA. Depths throughout the bay are relatively shallow, varying from 0–4 m with a mean of 2 m (MLLW); tides are semi-diurnal with a range of 0.8 meters [43]. In 2011, in the southeast portion of the bay, roughly 400 m from shore and 2.5 km east of the only oceanic inlet (40.857237° N, 72.450289° W), we selected a rectangular site measuring 250 m (parallel to shore) x 225 m (total area, 56,250 m^2^). No specific permission was required to work in this location or to passively quantify *Z*. *marina* coverage. Depths here ranged from 0.25–1.25 m MLLW. Surficial sediments were siliceous sands uniformly low in organic content (< 1% by loss on ignition at 500°C for 5 h, B. T. Furman unpubl.). Site orientation and boundaries were chosen: (1) to encompass the full meadow cross-section, (2) to minimize border contact with contiguous seagrass patches, (3) to eliminate the influence of light availability on seagrass distribution and (4) to capture sexual recruitment dynamics within a colonization phase meadow.

### Sexual Recruitment

**Fig 1:**
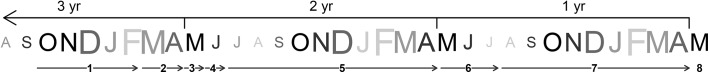
A life history timeline for sexual recruits censused via aerial photography. Monthly abbreviations are positively scaled to mean relative wave energy (RWE; J/m) and color graded to mean bottom-water temperature (proportion of days within optimal range). All means were calculated using the appropriate offset relative to spring census; annual marks describe the direction of offset. Bottom arrows denote significant life history stages during the inference period for sexual recruitment: i.e., (1) floral induction, (2) flower development, (3) anthesis, (4) embryo development and seed dehiscence, (5) seed bank, (6) seedling emergence, (7) patch development, and (8) photographic census.

Sexual reproduction in perennial *Z*. *marina* is thought to occur during the second season of growth and annually thereafter [44–46]; however, genet-wide flowering intensity has been shown to decline after the initial reproductive event [42]. In New York, flowering phenology is strongly correlated to temperature: floral induction occurs mid- to late-fall, primordial inflorescences appear in January at 0.5–3°C, anthesis occurs in mid-May at 15°C and fruit maturation is completed by the end of June at temperatures above 21°C [47, 48]. Seed dispersal covers three orders of magnitude (1s to 1,000s m), the distance and nature of which depend strongly on the form of the diaspora [28, 38, 49]. Negatively buoyant seeds diffuse roughly 5 meters via current- and wave-mediated rolling and saltational jumping [39, 42, 50], while the deposition of seeds from positively buoyant reproductive shoots and shoot fragments (i.e., spathes and rhapidia) can extend 10s to 1,000s of meters in a more or less spatially random fashion [38, 50, 51]. Dehisced seeds contribute to transient seed banks (1000s of seeds m^-2^) capable of germinating by their first fall, at temperatures below 20°C; however, yearlong (i.e., over winter) dormancy has been suggested [3, 34, 52, 53]. Correlative shoot demography data and December field surveys conducted at our site support the latter, indicating that a majority of seedlings emerge late May through early August, consistent with a spring germination (Furman et al. unpubl.). Genetic surveys have shown that isolated patches can begin with a single clone [49]. If so, seedling branching rates of between 2 and 12 x during the initial growth season [53, 54] and nominal patch spreading rates of between 13 and 46 cm y^-1^ [3, 54, 55] would delay detection of sexually recruited patches to, at minimum, the following spring. Based on these field and literature values, we have developed a working life history timeline (Fig 1) that tracks the inference period for sexual recruitment from the spring of observation backward nearly three years to the fall of floral induction.

To map sexual recruitment at our site, we gathered aerial photography from local, state and federal agencies via online access and personal communication. Annual geospatial images were obtained from the New York Statewide Digital Orthoimagery Program (NYSDOP; ground pixel resolution = 15 cm) and the USDA—Farm Service Agency’s National Agriculture Imagery Program (ground pixel resolution = 1 m; 2009 only) for the years 2006–2007, 2009–2010. In June of 2011, we began bi-monthly mapping of the study site by means of a custom-built, balloon-mounted camera (2011–2014, ground pixel resolution = 10–15 cm); see Furman et al. [42] for a full description of methods and available data. The images were photo-interpreted at an absolute resolution of 1:100 or greater using the ESRI ArcGIS software, ArcMap 9.2, and all seagrass patches were delineated manually as polygonal feature classes. The final sequence of high-resolution thematic habitat maps spanned 8 years, 2006–2014.

Sexual recruits were operationally defined as isolated, discontinuous patches that first appeared in spring-acquired images (March–May, when available). Recruits were distinguished from recently fragmented patches and areas recovering via surviving rhizomes by masking all locations at which seagrasses were recorded as previously present, nominally 2001 through the preceding December. Because the site was tidally swept and wave-exposed, we assumed patch formation through the re-settlement of up-rooted ramets to not occur (B. J. Peterson, person. obs). To minimize the confounding effects of naked seed transport from un-mapped areas, an internal 20-m buffer was placed over the site in an east-west direction. This was not necessary in the north-south orientation because the original site boundaries fully bracketed seagrass coverage. Recruits were divided into two classes following Furman et al. [42]: those falling within 6 m of seagrass mapped two springs prior (i.e., 0–6 m or naked seed dispersal, NSD) and those beyond (i.e., > 6 m or rafted seed dispersal, RSD). The numbers of recruits per year were then standardized by the availability (m^2^) of bare space within these distance classes at the time of seed dehiscence. Since NSD recruits were, by definition, produced locally, they were further normalized to the amount (m^2^) of seagrass coverage present during seed production. This area was calculated after the application of a internal 14-m, east west buffer (to the full site boundaries) to account for a maximum 6-m dispersal from maternal sources. Years for which coverage was unavailable at a two-year offset (2007 and 2010) were processed using distributions from the previous year.

### Relative Wave Energy

**Fig 2:**
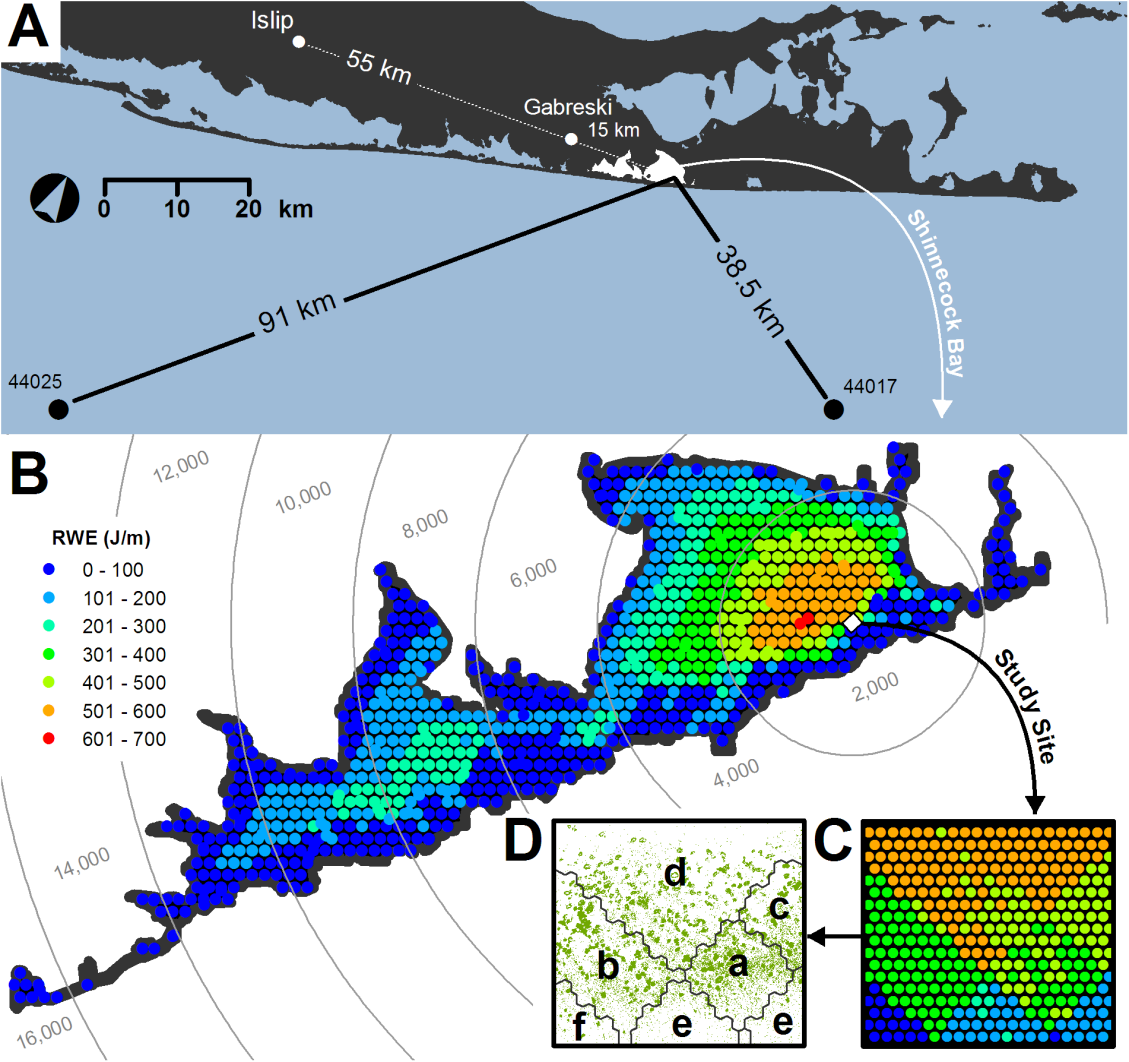
(A) Map of Shinnecock Bay, Long Island, New York in relation to sources of environmental data. (B) Mean wave energy conditions 2000 to 2014. Study site (56,250 m^2^) depicted as a white box. Gray lines delineate distance radii extending from site borders. (C) Mean relative wave energy (RWE) within the study site, 2000 to 2014. (D) Statistically significant multivariate zones (a-f) of RWE behavior, 2000 to 2014. Green polygons depict 2010 seagrass coverage.

Relative wave energies (RWE; J/m) were estimated using NOAA’s Center for Coastal Fisheries and Habitat Research Wave Exposure Model (WEMo v3.1). Hourly wind data were from Gabreski Airport in Westhampton Beach (USAF-WBAN station 744865–14719; Fig 2A) and bathymetric data were from the NOAA’s National Geophysical Data Center (Coastal Relief Model, ‘shinneco_9331’). WEMo uses the top 5% of recorded wind speeds to propagate simple linear waves along each of 32 lines of fetch, providing a useful measure of wave energy condition at any number of user-defined positions [56]. RWE values were generated for monthly intervals (2000–2014) at two scales of observation, bay-wide using a modified 200-m alternating grid of 964 positions (Fig 2B; S1 Table) and site-wide using a 12-m alternating grid of 369 positions (Fig 2C; S2 Table).

To characterize multivariate spatiotemporal patterns in wave energy, we first calculated Euclidean distance similarity matrices for each recruitment period between 2003 and 2014. This period was defined as May through the third previous August (Fig 1), encompassing all major life history stages for sexual recruits censused by our aerial surveys. We then generated a Spearman rank correlation matrix among years and conducted a second-stage analysis within each scale domain, both site and bay [57]. This approach has the unique benefit of circumventing issues of repeated measurement while still allowing for a full suite of multivariate comparison [57]. We used non-metric multidimensional scaling (nMDS) ordination, group-average agglomerative hierarchical clustering, and analysis of similarity (ANOSIM) routines to describe and quantify multi-year wave energy regimes at both the site and bay scales.

To reduce spatial complexity, wave energy data were summarized prior to MLR analysis. Based on the premise that rafted flower delivery would be a function of wave-mediated disturbance and distance from source, we binned bay-wide RWE measures into 2,000-m zones radiating from site boundaries (Fig 2B). This interval was chosen to approximately bracket regions of known seagrass coverage within the bay, and to provide a fair sampling of wave energy along the bay’s longest axis. To construct similar subdivision within our study site, group-average agglomerative hierarchical clustering and nMDS analysis of the full RWE time-series (January 2000 to April 2014) was used to delineate zones of statistically similar decadal behavior. During this process, spatially coherent clusters were favored. Mean, maximum and standard deviation estimates for site-wide, monthly wave energy conditions were also examined.

### Water Temperature

On-site temperature records were unavailable for the required duration (2004–2014), and so an effort was made to estimate historical bottom-water temperatures from more accessible meteomarine and atmospheric variables (e.g., air temperature, wind chill, sea-surface temperature). Within our study site, we directly measured bottom-water temperatures for 2012–2014 at 15-min intervals using HOBO Onset light and temperature dataloggers anchored 10 cm from the sediment-water interface. In June of 2012, we placed sensors at the corners and center of the site, and in March of 2013, we expanded this to include 13 additional dataloggers. Daily site-wide means were calculated and, to dampen sensor fluctuations, the time-series was smoothed using a 7-pt running average. Comparable sea-surface temperatures were obtained from the two closest NOAA National Data Buoy Center stations, 44017 and 44025 (Fig 2A). Oceanic datasets were averaged to produce a complete 2004–2014 daily mean time-series. Associated air temperature (°C) and wind speed (m/s) data were acquired from Westhampton Beach Gabreski Airport (Fig 2A). Using MLR, we modeled the temperature offset between site and oceanic conditions using a fully saturated model with air-sea differential (°C), wind chill (W m^-2^) and wind speed (m/s, mean conditions during the previous 3 days) as predictor variables (adj-R^2^ = 0.7134, P<0.001; S3 Table). The relationship was then hind-cast to generate a 2004–2014 time-series of daily mean site temperatures. These data were crosschecked using monthly bay-wide (11 sites) temperature ranges recorded by the Suffolk County Department of Health Services. Model performance was acceptable, placing projected temperatures within 1 degree of observed values in 93% of cases (N = 107).

Monthly summaries were calculated by dividing the number of days mean conditions fell within each of 6 temperature zones (<0°C, <5°C, <10°C, 10–20°C, >20°C and >25°C) by the number of days available within each month. We based these thresholds on literature values for cold- and heat-related temperature stress, a well-known control on *Z*. *marina* productivity and distribution [19, 21, 58–60]. Threshold exceedance values for three months (January 2009, September—October 2011) were linearly interpolated due to missingness.

### Atmospheric Variables

Because of the shallow, enclosed nature of Shinnecock Bay, we chose to include monthly estimates of atmospheric condition as potential correlates with on-site disturbance. Mean monthly rainfall was calculated using precipitation data obtained from the Long Island MacArthur Airport in Islip, NY (USW00004781; Fig 2A). Mean wind directions were derived from Gabreski Airport observations after extraction of continuous orthogonal components (i.e., ‘northness’ and ‘eastness’) following the methods of Zar 1999 [61, 62]. Mean monthly wind speeds (m/s) from the same dataset were averaged after removal of ‘variable’ and ‘calm’ observations.

### Multiple Linear Regression Analysis

To mitigate issues associated with data mining (i.e., ghost degrees of freedom and spurious temporal correlations) we employed a mixed approach to variable selection, integrating both site and biological knowledge with statistical information at each phase of the process. Because we began the investigation with few data regarding the critical time-period for sexual recruitment–that is, which life history stages were the most vulnerable to ecophysiological stress–we adopted an exhaustive time-lagging procedure. All potential predictor variables (N = 27; S4 Table) were screened for linear relationships with each of the dependent variables (i.e., NSD and RSD recruitment) using Pearson correlations; (1) monthly offsets extending backward one year, (2) a sequence of expanding durations of up to one full previous year, (3) full 24- and 36-month means, and (4) 3-month means extending back 3 years were evaluated. Those exhibiting a significant relationship with either or both response variables (P<0.05) were visually assessed for outlier influence and dispersion along the abscissa. Multicollinearity among the remaining variables was quantified by hierarchical clustering of absolute transformed Pearson correlation matrices, one matrix per response variable. Co-linear groups were set at a threshold of 0.70 [54] and culled to a maximum of 4 per group based on ecological interpretability and statistical similarity (i.e., redundancy). All potential combinations of uncorrelated variable sets were then entered into the ‘regression with empirical variable selection’ or REVS procedure following the methods of Goodenough et al. 2012 [63]. This method uses an all-subsets approach, implemented through the ‘leaps’ package for R, to investigate and rank predictor importance so as to empirically order a manual stepwise regression [64, 65]. The resulting candidate models were sorted by adj-R^2^ and diagnostically tested for multicollinearity (variable inflation factor or VIF), model complexity (Akaike’s Information Criterion or AIC), model bias (Mallows’ C_p_ statistic), standardized residual normality (Shapiro-Wilk’s test), homogeneity of variance (Breusch-Pagan test), residual independence (Durbin Watson statistic), and outlier influence (Cook’s distance). To account for small sample size (N = 7) and to address issues of over-fitting, we further evaluated candidate models using a combination of second-order AIC (AIC_C_), AIC_C_ weights and leave-one-out cross-validation [LOOCV; 66]. The best model for each recruitment type (NSD and RSD) was selected based on (1) diagnostic performance, (2) parsimony of biological explanation, (3) future repeatability (i.e., independent variables that were statistics of location were preferred over those of dispersion) and (4) number of implicated life history stages. Response variables were log-transformed to meet assumptions of normality. All univariate analyses were conducted on the open-source R-Package (v2.14.1), and all multivariate analyses were performed using PRIMER (v6.1.15).

## Results and Discussion

### Sexual Recruitment

Seven snapshots of meadow development were recorded during the 8-year observation period. During this time, *Z*. *marina* coverage increased from 4.8 to 42.7% of the mapped area. Gains were stepwise in pattern and attributable to annual recruitment of new patches–all less than 4 m^2^ at the time of first observation–followed by slow centrifugal growth and coalescence. Recruitment was not constant over time, however, varying across 2 orders of magnitude from 13 to 4,894 patches yr^-1^ with a mean (± 1 s.d.) of 1,076 ± 1,716 patches yr^-1^. Naked seed recruits (0–6 m) ranged 11 to 2,978 (722 ± 1,025) patches yr^-1^, while rafted seed recruits (> 6 m) were less abundant at between 2 and 1,916 (353 ± 706) patches yr^-1^. Minima and maxima for both recruitment types took place in 2009 and 2010, respectively, with higher numbers of recruits observed post-2009.

### Wave Energy Regimes

**Fig 3:**
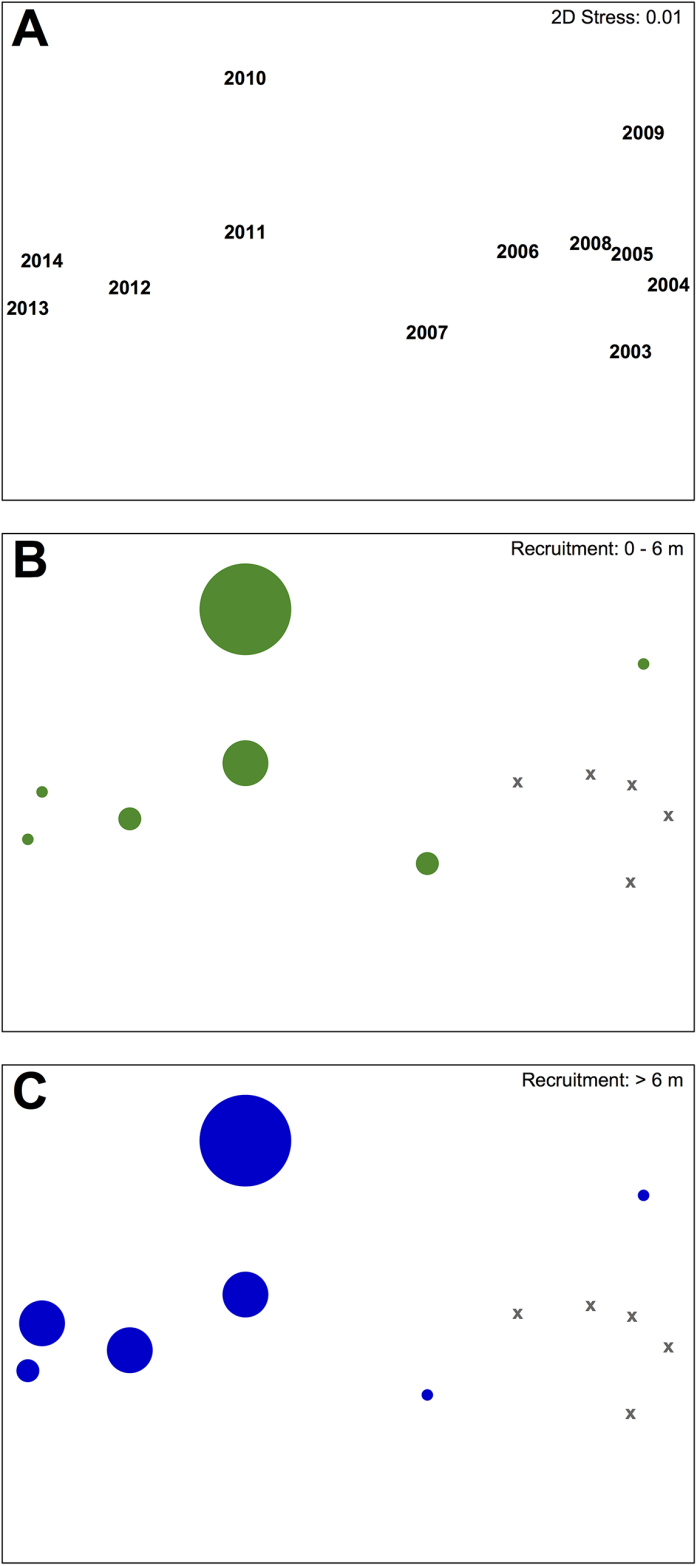
Second-stage nMDS of monthly relative wave energy (RWE) within Shinnecock Bay, 2003 to 2014. Each symbol represents an RWE sequence during the recruitment period, defined as May of observation through the third previous August. Symbols are shown by (A) yearly recruitment event and superimposed with (B) NSD and (C) RSD recruitment. X’s reference years for which no recruitment estimates were made.

**Fig 4:**
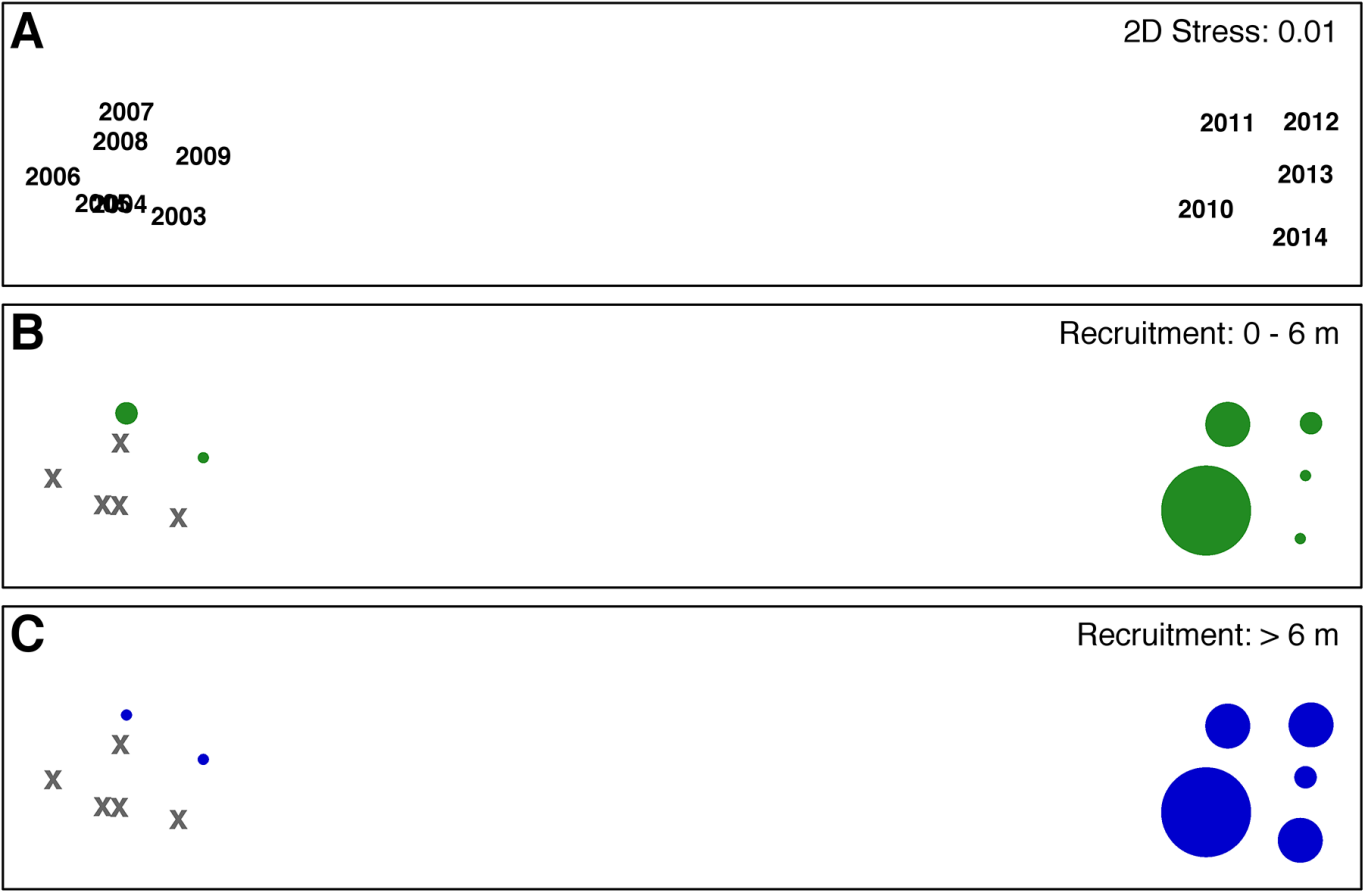
Second-stage nMDS of monthly relative wave energy (RWE) within the study site, 2003 to 2014. Each symbol represents an RWE sequence during the recruitment period, defined as May of observation through the third previous August. Symbols are shown by (A) yearly recruitment event and superimposed with (B) naked seed dispersal (NSD) and (C) rafted seed dispersal (RSD) recruitment. X’s reference years for which no recruitment estimates were made.

nMDS ordination of bay-wide conditions suggested two distinct clusters of recruitment-relevant RWE, one covering 2004–2009 and another 2010–2014 (Fig 3A). ANOSIM revealed these groups to be highly significantly different (global R = 0.978, P = 0.001) despite an among-group correlation coefficient of 0.96. Interestingly, both anomalous years (2009 and 2010) were somewhat isolated within their respective clusters, each along the same axis in the 2-d representation (stress = 0.01). Graphical overlays of NSD and RSD recruitment rates were consistent with the view that multivariate sequences of bay-wide RWE mirrored long-term patterns in sexual recruitment, and even appeared to structure gradients of recruitment success within each long-term regime (Fig 3B and 3C). Similar patterns were found for wave energies estimated within the study site, as Spearman rank-based Mantel tests between bay- and site-wide RWE resemblance matrices (RELATE procedure in PRIMER) were highly correlated (ρ = 0.858, P = 0.001). Likewise, nMDS and hierarchical clustering analyses described identical clusters (2004–2009 and 2010–2014) that were also significantly different (global R = 1, P = 0.001; Fig 4A). Not surprisingly, patterns at this small spatial grain were more correlated with each other (ρ = 0.990) and less suggestive of gradients when overlain with sexual recruitment rates (Fig 4B and 4C). We interpreted this to be a possible artifact of WEMo model performance, whereby the resolution of bay-wide bathymetric data (cell-size = 83.2 x 83.2 m) was more suited to linear wave propagation at the landscape scale, providing a richer treatment of small differences in wind conditions when modeled over the same time period. For this reason, we favored RWE-based predictors drawn from the bay scale when selecting among potential independent variables (see ‘Variable Selection‘).

Unfortunately, no specific test for group cohesion exists for second-stage analysis; however, successive similarity percentage (SIMPER) tests run on transposed component matrices (i.e., by recruitment period at the site-scale) and temporal patterns of median RWE values indicated that the nMDS clusters were a product of slightly relaxed wave energies during the 2010–2014 regime. We posit that such changes, which would amount to a reprieve for vulnerable seeds and seedlings, could be particularly impactful at our study site, as long-term (2000–2014) averages place it within the highest wave energy zone of the bay (Fig 2B).

nMDS ordination and hierarchical clustering of long-term (January 2000 through April 2014) RWE patterns at the site level identified 6 roughly contiguous zones of wave energy (hereafter, a-f; Fig 2C and 2D). One-way ANOSIM confirmed these to be statistically distinct (global R = 0.998, P = 0.001) with extremely high pair-wise R-values, all 0.993 to 1. Mean conditions within zones a-f over the 178-month period (January 2000 to October 2014) were 399.2 ± 2.93, 350.51 ± 11.57, 454.88 ± 3.71, 507.34 ± 8.25, 195.79 ± 3.42 and 24.62 ± 9.87 J/m, respectively, or d, c, a, b, e and f in descending order (Fig 2C and 2D). As would be expected under scenarios of disturbance-control, seed-borne patches during the largest recruitment event, in 2010, were aggregated within the higher energy zones (i.e., a, c and d) (Fig 2D), suggesting that quiescent conditions may have facilitated recruitment success.

### Multiple Linear Regressions

#### Variable selection

Significant Pearson correlations between environmental variables and log-transformed NSD and RSD recruitment estimates were found for 152 time-lagged summaries: N = 48 and 105, respectively. Outliers or biphasic groupings unduly influenced a large number of these relationships, leaving only 23 (NSD) and 30 (RSD) viable predictors for multicollinearity assessment. Both lists were further pared to eliminate statistical and temporal redundancy, which yielded 12 variables clustered into 4 groups for the NSD model, and 9 variables into 3 groups for the RSD model. Counter to expectations–we had anticipated disturbance of distant flowers to control RSD recruitment–both sets of significant correlates were composed of nearly equivalent proportions of wave energy (56–8%) and site-specific (33%) products. NSD predictors were also temporally clustered, corresponding to floral induction, seed bank and patch development life history stages (Fig 1). Eight of these varied negatively with recruitment, with 3 of the 4 positive associations linked to seed bank conditions during the fall and winter. In slight contrast, (1) only a single RSD-associated variable (mean maximum RWE during the second previous August to October) was positively related to recruitment, (2) nearly all of the viable predictors (75%) implicated conditions during patch development, and (3) a fourth life history stage was added, flower growth and maturation (negatively correlated to 0°C exceedance during February to April).

While it is tempting to interpret these patterns mechanistically, it is important to recognize that independent variable associations are purely descriptive and, as such, are unable to define causality within the system [26]. Nevertheless, the correspondence of time-lagged disturbance measures to particularly vulnerable life history stages, such as seed banks and initial clonal growth, is encouraging, and provides specific targets for future manipulative work. For example, understanding the physiological controls of floral induction [47] and seed germination [34], as well as the precise mechanisms and seasonality of small patch mortality [3, 67], are all tractable research questions.

#### Model selection

After duplicate removal, the modified REVS procedure yielded 31 candidate NSD models; 6 of these failed diagnostic testing due to multicollinearity, model bias and residual non-normality. The remaining models explained between 77 and 99.8% of the variation using 2 to 4 predictors. Although we recognize that ensemble predictions (i.e., the use of multiple models) often provide important insights into the scale and nature of forecast uncertainty, and have been used successfully to describe *Z*. *marina* coverage in the past [16], because the candidate models often shared one or more predictor variables and were all of the same statistical framework, we chose instead to select a single, or best, model for each recruitment type. This simplified initial predictions and interpretation. Future implementation of these methods, however, may wish to query multiple models as some portion of the underlying statistical patterns may have proven falsely positive.

**Fig 5:**
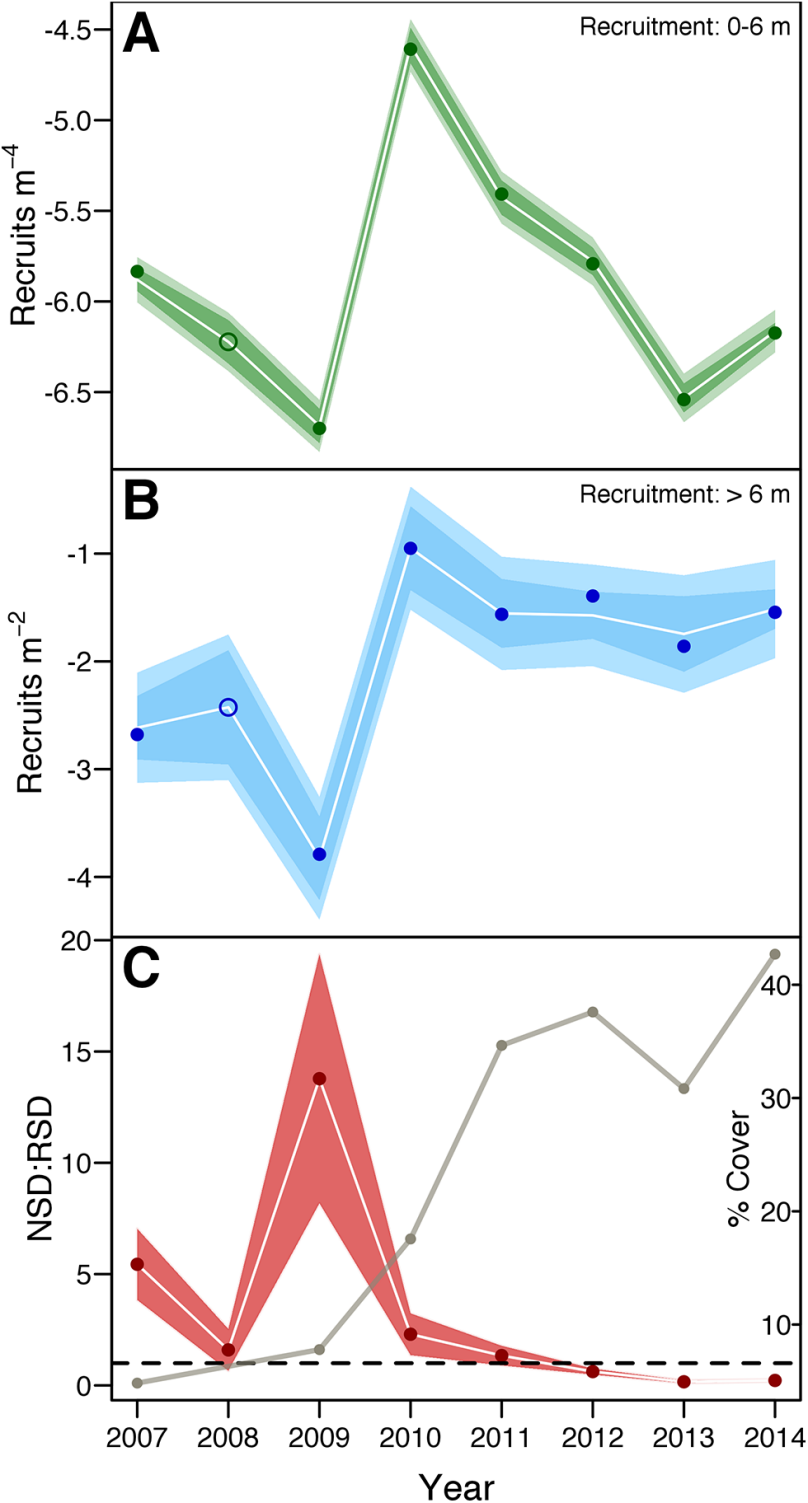
Sequence of modeled (A) naked seed dispersal (NSD) and (B) rafted seed dispersal (RSD) recruitment. Dark shaded regions depict 95% confidence intervals, lighter regions 95% prediction intervals. White lines follow model predictions. Filled circles locate actual values and open circles model predictions. The relative importance of NSD to RSD recruitment (C) as calculated using standardized, fitted data (filled, red circles) compared to the proportion of the study site covered by seagrass (tan line). Red shaded region shows the propagated error for each NSD:RSD estimate.

Based on statistical diagnostics and ecological considerations, the most promising NSD model additively used (1) mean 10°C exceedance during the previous September to November (‘10C_p1yNOS’), (2) mean rainfall during the second previous November to January (‘rain_p2yJDN’), and (3) mean wind eastness during the preceding 3-yr period (‘eastness_p36mo’)–taking the form: log(NSD) = -8.72710 * 10C_p1yNOS + 0.00139 * rain_p2yJDN + 8.48952 * eastness_p36mo—4.37839 (adj-R^2^ = 0.998, P < 0.001; Fig 5A). This model was ranked 8^th^ based on AIC_C_ weights; however, it out-performed all other candidates in LOOCV and had the lowest un-adjusted AIC value. Interestingly, despite the large number of available inverse relationships, two of the three independent variables were positively correlated with NSD recruitment, and none of them were estimates of wave energy. The latter suggests that once seagrass patches become established, subsequent NSD recruitment may not be strongly limited by wind waves. Whether this is a function of greater densities of NSD seed banks [39, 42] or some hydrodynamic modification afforded by proximate seagrass coverage [56] remains unclear.

Cold-induced torpor (< 10°) during early patch development was indicated as a negative control on NSD recruitment. This could be a product of clone mortality or merely a reduction in vegetative propagation, leaving patches too small to identify in spring-acquired imagery. As the balloon-mapping program continues, fall and winter maps will help to resolve this issue through the identification of late-arriving recruits, especially during anomalously cold years. Future, manipulative work should examine the physiological mechanism behind this relationship. Our expectation is that metabolic depression acts directly to limit branching frequency and carbon storage; however, likely covariates, such as reduced solar irradiance and bioturbation rates cannot be discounted.

We interpret the influence of rainfall as a source of freshwater to extant seed banks, with direct effects on germination rates. Similar effects of precipitation and terrestrial runoff have been suggested for Pacific populations of *Z*. *marina* [68]. If not a direct germination cue, rainfall rates can also co-vary with meteomarine conditions promoting temperature stratification in surficial sediment layers or the scarification of seed-coats (i.e., intermittent storm activity not captured by WEMo), as both are known to be drivers of seed germination [34].

For RSD recruitment, the REVS procedure generated 14 models. Poor fit disqualified 3 of them from further consideration, leaving 11 candidate models explaining 80 to 98% of the variance. The majority of predictors comprising these models were wind or RWE-related (78%), as were all three independent variables of the best model: (1) mean RWE within the 14,000- to 16,000-m distance class during the previous November to April (‘d16000_pAprToNov’), (2) mean wind speed during the previous May (‘mWindSpd_pMay’), and (3) mean RWE in the 0- to 2,000-m distance class during the previous February (‘d2000_pFeb’). The model: log(RSD) = -0.23381 * d16000_pAprToNov—0.54899 * mWindSpd_pMay—0.00107 * d2000_pFeb + 8.07992, was ranked 2^nd^ in AIC_C_ weights, 1^st^ in LOOCV performance and explained 98.1% of the variation (P = 0.002). Importantly, all three of these terms referenced wave energy characteristics experienced during early patch formation, all negative correlates with recruitment. This is consistent with the notion that isolated patches remain vulnerable to hydrodynamic forces for some time after initial formation [3, 54]. Whether this was due to burial [67], scour [56] or breakage of ramets is unclear from these findings; however, some stability appears to be conferred by the presence of adjacent individuals [2, 69], as the loss of established patches (~ 1 m^2^) was not regularly observed. Similar findings have been reported for small patch dynamics in Danish waters, where a size threshold of 32 ramets was identified as a necessary buffer against physicochemical disturbance [3].

#### Model predictions

Limited by the available time-series, we were unable to validate either model on new coverage data. However, projections for the un-measured 2008 recruitment event fit *a priori* expectations based on known changes in landscape configuration, and together provide a sensible picture of sexual recruitment dynamics over the modeled period (Fig 5A and 5B). Both the NSD and RSD models yielded values intermediate to 2009 and 2010 estimates. Admittedly, this approach was slightly confounded by the duplication of seagrass and distance class information, although we feel model success here reflects positively on our capability to process future data.

The relative contributions of NSD and RSD recruitment to our study site were assessed using the fitted data by standardizing model predictions to fixed seagrass coverage and available space values (Fig 5C). Temporal patterns of NSD:RSD recruitment and their propagated errors showed that NSD recruitment or the dispersal of naked seeds from source plants was comparatively more important than RSD or the dispersal of rafted seeds during the 2007 to 2011 period. This reversed after 2011, in rough parallel with rising seagrass coverage (Fig 5C). One interpretation of this pattern is that long distance dispersal of rafted flowers was relatively unimportant to space acquisition at dispersal distances of 2,000 to 4,000 m, or roughly the distance from the study site to the next largest meadow prior to 2010, and only after seagrasses began to densely colonize the southeastern shoreline did RSD recruitment contribute significantly to meadow expansion. If true, this means that RSD kernels operate largely below 2,000 m, a hypothesis that is testable using current genetic methods. Alternatively, this could have been an artifact of sharp reductions in available space in RSD distance class, particularly the loss of distances greater than 20 m. By increasing the relative contribution of more proximate recruits, RSD estimates could have been conflated with limited numbers of NSD recruits beyond 6 m–a possibility supported by empirical estimates of seed dispersal distance [39, 42].

## Conclusions

Ecophysiological stress and physical disturbance are known agents of change in seagrass systems, capable of spatially structuring meadows through a combination of direct biomass removal and recruitment limitation [10, 19, 56]. In the present study, we selected a light-replete study site with seasonal extremes of temperature and wave energy to model environmental effects on sexual recruitment over an 8-year period. Two multiple linear regression models were developed, one described the annual recruitment of seed-borne patches arising from the diffusive flux of naked seeds (NSD) and, another, from seeds deposited by rafted flowers (RSD).

Results indicated that sexual recruitment varied as a predictable function of wind, temperature and wave energy, with long term multivariate patterns in wave energy corresponding to periods of rapid colonization within our site. Notably, no comparable multivariate patterns were found for combinations of atmospheric condition (i.e., rainfall, wind speed, wind direction or air temperature). Univariate correlational patterns with time-lagged climatic summaries consistently showed floral induction, seed bank and small patch developmental periods to be most vulnerable to disturbance. Of these, seedling survival was probably the most important, as seedling safe-site availability has been shown to control aspects of space acquisition during periods of rapid expansion and recovery [46, 70], and physical forces are known to play significant roles in seedling survival and resultant bed morphology [27, 56, 71].

For this modeling approach to have broader spatiotemporal applicability (i.e., forecasting power), sufficient environmental variation must have been sampled during its initialization. That is, meadow responses to a full range of climatic conditions need to have been incorporated into the model for it to then accurately predict recruitment when presented with new data. At present, there is no way to know if this has occurred; however, (1) normal temperate-latitude cycles of temperature and wave energy were observed, (2) the time-series included at least one anomalously warm year, 2012, (3) a strong hurricane impacted the system, Hurricane Sandy, in 2012, and (4) a wide range of sexual recruitment was documented, including the largest event recorded since 2001. Future application of the models developed in this study should apply caution, however, if input data fall outside the ranges reported in Table 1, as the underlying correlational structure may no longer be valid.

**Table 1:** Observed monthly ranges for environmental variables, 2004 to 2014.

Abbrev.	Description	Jan	Feb	Mar	Apr	May	Jun	Jul	Aug	Sep	Oct	Nov	Dec
SAV	pct. of site covered by seagrass (%)	-	-	-	-	4.83–42.70	-	-	-	-	-	-	-
25C	prop. of days mean daily temp. exceeded 25°C	0.00	0.00	0.00	0.00	0.00	0.00	0.00–0.52	0.00–0.52	0.00–0.13	0.00–0.07	0.00	0.00
20C	prop. of days mean daily temp. exceeded 20°C	0.00	0.00	0.00	0.00	0.00–0.10	0.10–0.60	0.90–1.00	0.87–1.00	0.18–0.90	0.00–0.33	0.00	0.00
optimal	prop. of days mean daily temp. was optimal	0.00–0.16	0.00	0.00–0.19	0.03–0.60	0.77–1.00	0.40–0.90	0.00–0.10	0.00–0.13	0.10–0.82	0.53–1.00	0.50–0.93	0.03–0.35
10C	prop. of days mean daily temp. was less than 10°C	0.84–1.00	1.00–1.00	0.81–1.00	0.40–0.97	0.00–0.23	0.00	0.00	0.00	0.00–0.07	0.00–0.14	0.07–0.50	0.65–0.97
5C	prop. of days mean daily temp. was less than 5°C	0.32–0.92	0.48–0.96	0.16–0.90	0.00–0.23	0.00	0.00	0.00	0.00	0.00	0.00	0.00–0.07	0.06–0.68
0C	prop. of days mean daily temp. was less than 0°C	0.00–0.32	0.00–0.18	0.00–0.13	0.00	0.00	0.00	0.00	0.00	0.00	0.00	0.00	0.00
rain	monthly precipitation (tenths of mm)	553–1065	320–1580	253–2391	389–1852	380–1494	314–2241	532–1655	67–2939	333–1896	56–3574	486–1160	545–2242
eastness	magnitude of the easterly wind component (unitless)	-0.51–-0.11	-0.59–-0.14	-0.43–0.11	-0.37–0.17	-0.29–0.21	-0.33–0.19	-0.44–-0.00	-0.24–0.02	-0.26–0.18	-0.34–0.04	-0.40–0.05	-0.51–-0.17
northness	magnitude of the northerly wind component (unitless)	0.02–0.46	0.04–0.39	-0.04–0.45	-0.25–0.20	-0.35–0.16	-0.51–-0.01	-0.54–-0.13	-0.40–0.01	-0.19–0.14	-0.18–0.31	-0.18–0.48	-0.03–0.37
mWindSpd	mean wind speed (m/s)	4–5	5–6	4–6	4–5	4–5	3–4	3–4	3–4	4	4–5	4–5	4–6
mRWE	mean RWE (J/m) at the site (N = 369)	318–603	428–1057	292–967	232–635	165–763	119–424	51–224	49–195	69–262	120–652	174–677	255–794
sdRWE	standard deviation of RWE (J/m) at the site (N = 369)	93–227	131–465	99–383	55–208	42–278	23–125	13–58	13–54	13–69	28–243	44–249	65–296
mxRWE	maximum RWE (J/m) at the site (N = 369)	425–853	605–1556	402–1376	283–846	220–1065	141–549	74–283	69–290	88–367	146–924	252–941	321–1115
a_mRWE	mean RWE (J/m) in Zone A of the site (N = 48)	309–624	381–1126	279–1023	233–653	141–792	119–424	43–221	43–195	62–231	120–689	152–704	251–837
b_mRWE	mean RWE (J/m) in Zone B of the site (N = 75)	280–520	405–847	268–779	221–581	149–663	123–400	44–207	42–189	69–254	113–541	175–586	237–655
c_mRWE	mean RWE (J/m) in Zone C of the site (N = 30)	355–697	451–1291	333–1169	253–733	176–905	122–471	59–252	57–208	73–262	133–785	168–795	283–952
d_mRWE	mean RWE (J/m) in Zone D of the site (N = 153)	397–793	543–1450	377–1294	272–802	209–994	131–523	63–269	60–236	78–319	140–856	209–885	305–1044
e_mRWE	mean RWE (J/m) in Zone E of the site (N = 46)	195–257	235–384	134–350	135–313	113–304	86–248	38–160	36–171	59–203	95–268	139–290	190–340
f_mRWE	mean RWE (J/m) in Zone F of the site (N = 17)	18–28	22–34	21–32	23–32	20–31	18–28	10–24	16–27	18–25	16–31	20–31	23–33
d2000	mean RWE (J/m) 0–2000 m from site (N = 228)	266–488	411–876	333–758	186–495	143–590	92–328	70–198	90–203	95–287	101–567	181–502	225–631
d4000	mean RWE (J/m) 2000–4000 m from site (N = 263)	147–270	175–458	159–435	126–293	96–376	62–190	37–198	52–261	87–352	44–634	121–477	115–345
d6000	mean RWE (J/m) 4000–6000 m from site (N = 107)	67–106	78–195	60–148	52–124	51–145	32–88	17–80	23–113	34–117	24–204	61–174	55–135
d8000	mean RWE (J/m) 6000–8000 m from site (N = 165)	119–171	134–253	119–226	78–167	62–177	47–117	28–87	38–94	47–133	37–218	88–186	88–199
d10000	mean RWE (J/m) 8000–10000 m from site (N = 113)	88–138	101–242	85–209	67–144	59–189	36–105	22–95	27–125	44–172	29–277	77–226	65–175
d12000	mean RWE (J/m) 10000–12000 m from site (N = 70)	43–79	54–131	48–137	41–77	31–126	18–61	12–60	16–82	27–123	15–177	36–138	35–111
d14000	mean RWE (J/m) 12000–14000 m from site (N = 9)	7–11	8–20	6–19	8–19	6–15	3–9	2–9	3–13	5–15	3–18	7–15	7–16
d16000	mean RWE (J/m) 14000–16000 m from site (N = 9)	17–28	24–62	20–50	15–35	11–40	7–21	4–14	7–13	7–17	7–44	11–32	16–43

Although spatially explicit, discrete (stage-based) or continuous (differential) population growth models are best suited to describing landscape dynamics, it may be sometime before working process models of seagrass recruitment become available [26]. Until then, statistical forecasting tools such as the ones described above could be used in conjunction with more common habitat distribution models to better understand the drivers and temporal patterns of coverage change. This is particularly true for areas of commission error, where habitat models predict seagrass presence but none is currently found. With so much of the global habitat space lost or degraded, these regions are increasingly common. Methods such as ours could be used to predict colonization trajectories for areas of commission error proximate to extant seagrasses, and to augment success criteria by quantifying coverage expectations for management zones and restoration projects that lack appropriate reference locations, allowing coastal managers to track conservation and mitigation progress in real-time. To our knowledge, this study represents the first attempt to relate relative wave energy to seagrass recruitment success in a temporally explicit manner, at a scale of action necessary for effective coastal management.

## Supporting Information

S1 File Overview of sexual recruitment model. (PDF)

S1 Table Monthly bay-wide relative wave energy (RWE) estimates used to characterize wave energy regimes. (XLSX)

S2 Table Monthly site-wide relative wave energy (RWE) estimates used to characterize wave energy regimes. (XLSX)

S3 Table Raw data used to model daily mean bottom-water temperatures. (XLSX)

S4 Table Raw data used in the sexual recruitment models. (XLSX)
