# Supplementary material for: Sexual Recruitment in Zostera marina: Progress toward a Predictive Model
Source: PLoS One. 2015 Sep 14;10(9):e0138206. doi: 10.1371/journal.pone.0138206 (PMC4569585; doi:10.1371/journal.pone.0138206)
Supplement: S1 File — (PDF) [file pone.0138206.s001.pdf]

Sexual Recruitment Model

Rationale:

1. Flowering has not changed over time
2. Light limitation is not an issue
3. Competition is not an issue
4. Wave exposed (WEMo; Slide 11)
5. Temperature stressed (Slide 2)

Sexual Recruitment Model

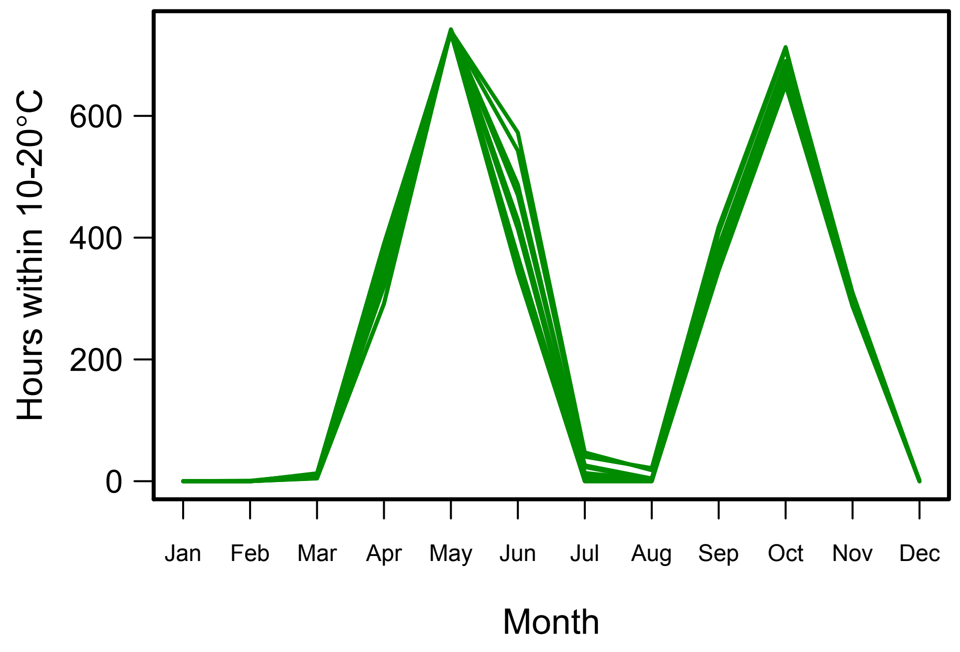

13 HOBO temperature loggers
15-min readings
1-year deployment (2013)
Optimal range: 10 – 20°C

H_A: Inter-annual variability in sexual recruitment would be a predictable function of environmental stress

Multiple Linear Regressions

Available seagrass data:

1. 2006 – 2014 (8 years)
2. Missing 2008

Dependent variables:

Dispersal distance ranges based on Furman et al. 2015

1. Rafted seed dispersal (spatially random) > 6 m

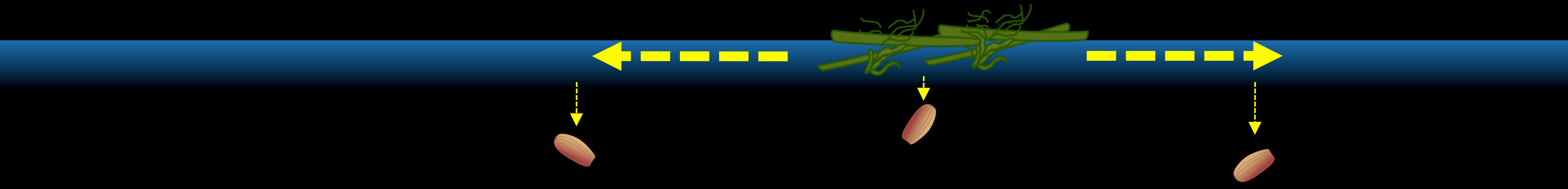

2. Naked seed dispersal (diffusive process) 0 – 6 m

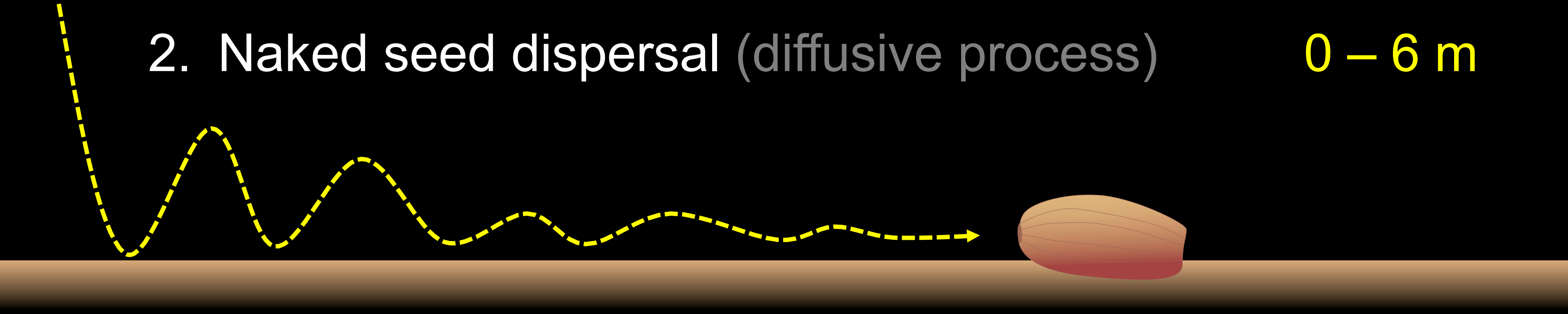

Multiple Linear Regressions

Calculating recruitment:

1. Naked seed dispersal — 0 – 6 m
2. Rafted seed dispersal — > 6 m

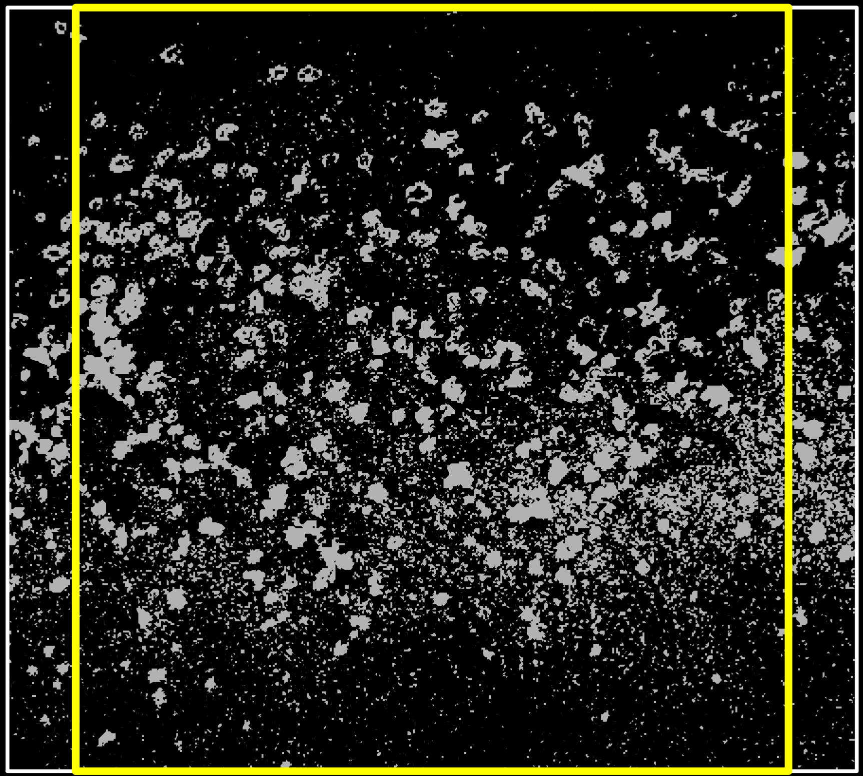

20-m buffer

Multiple Linear Regressions

Calculating recruitment:

1. Naked seed dispersal 0 – 6 m
2. Rafted seed dispersal > 6 m

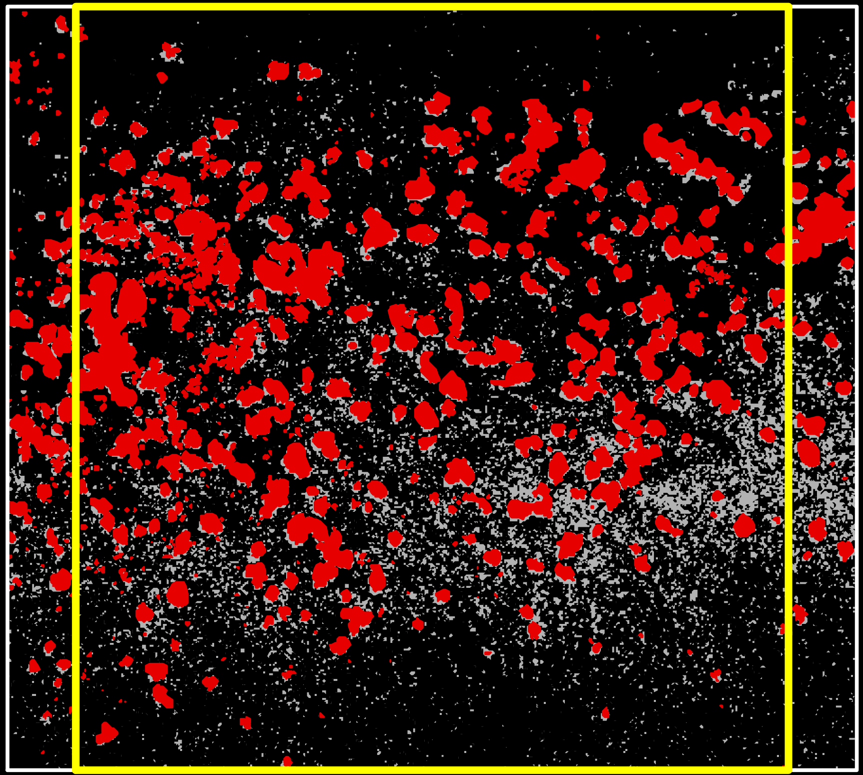

20-m buffer
Remove historical coverage

Multiple Linear Regressions

Calculating recruitment:

1. Naked seed dispersal 0 – 6 m
2. Rafted seed dispersal > 6 m

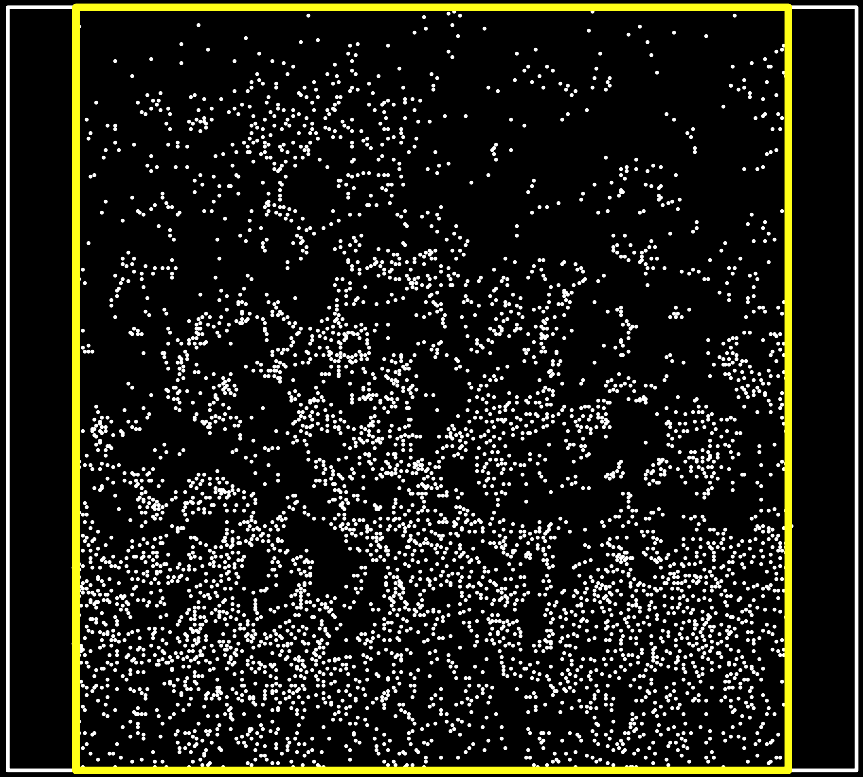

20-m buffer
Remove historical coverage
Locate recruited patches

dots = patches

Multiple Linear Regressions

Calculating recruitment:

1. Naked seed dispersal — 0 – 6 m
2. Rafted seed dispersal — > 6 m

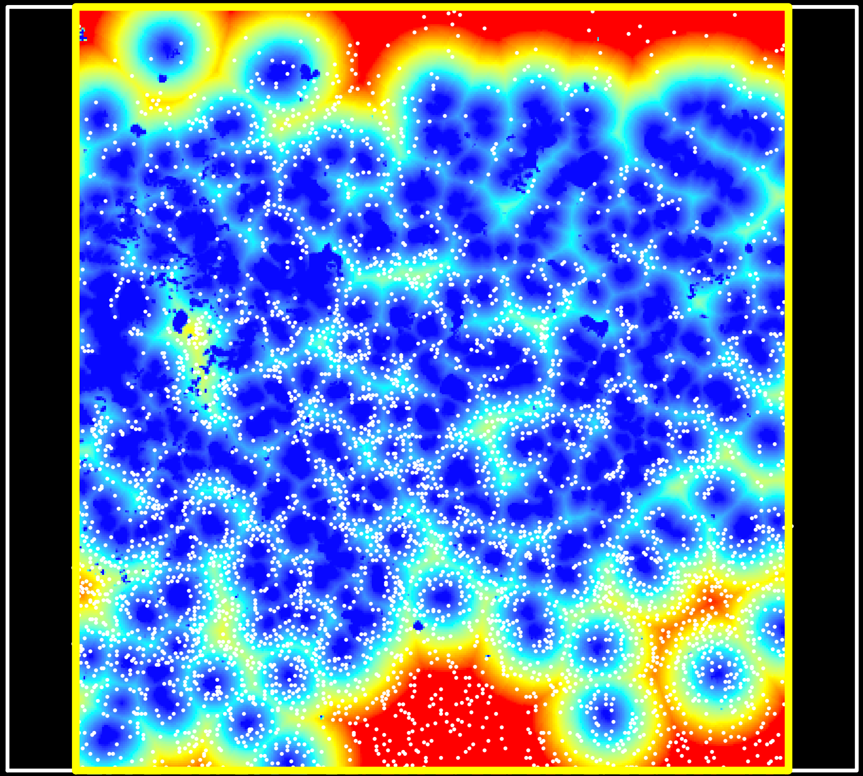

20-m buffer
Remove historical coverage
Locate recruited patches
Calculate distance to spring coverage
Standardize to available space

dots = patches

Multiple Linear Regressions

Calculating recruitment:

1. Naked seed dispersal 0 – 6 m
2. Rafted seed dispersal > 6 m

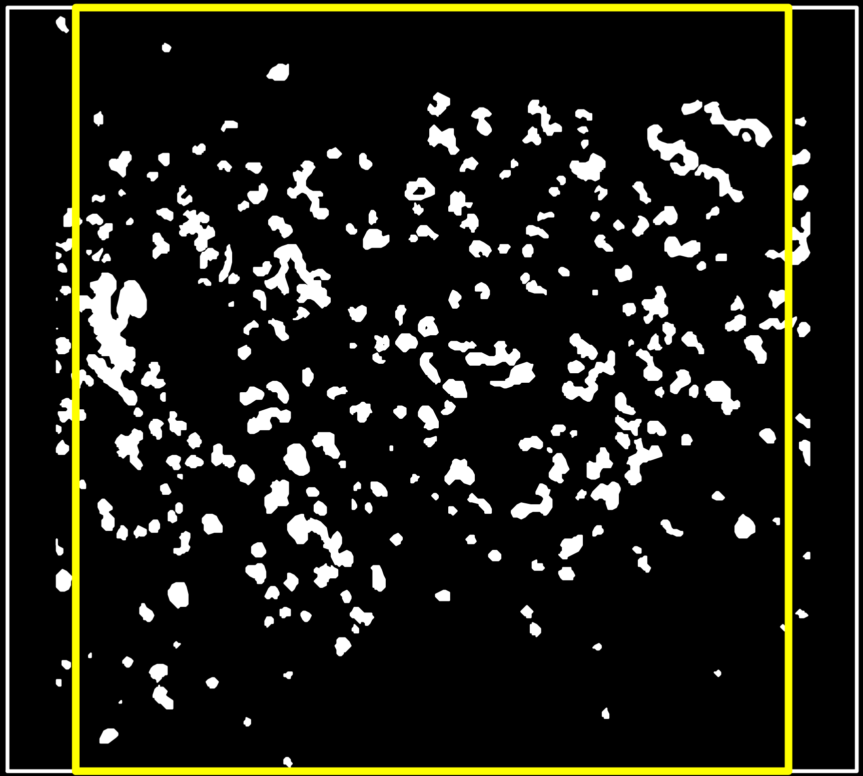

20-m buffer
Remove historical coverage
Locate recruited patches
Calculate distance to spring coverage
Standardize to available space

Naked: Standardize to available coverage

Multiple Linear Regressions

Predictor variables:

1. Rainfall from Islip Airport
2. Wind speed & direction from Gabreski Airport
3. Water Temperature

   Modeled from NOAA buoy (2) and Gabreski Airport data

   adj-$R^2 = 0.71$, $P<0.001$

Monthly Summaries: # d mean > threshold / # d in month

< 0°C	< 5°C	< 10°C	10 -20°C	> 20°C	>25°C

4. Wave Energy

   NOAA’s Center for Coastal Fisheries and Habitat Research Wave Exposure Model (WEMo v3.1)

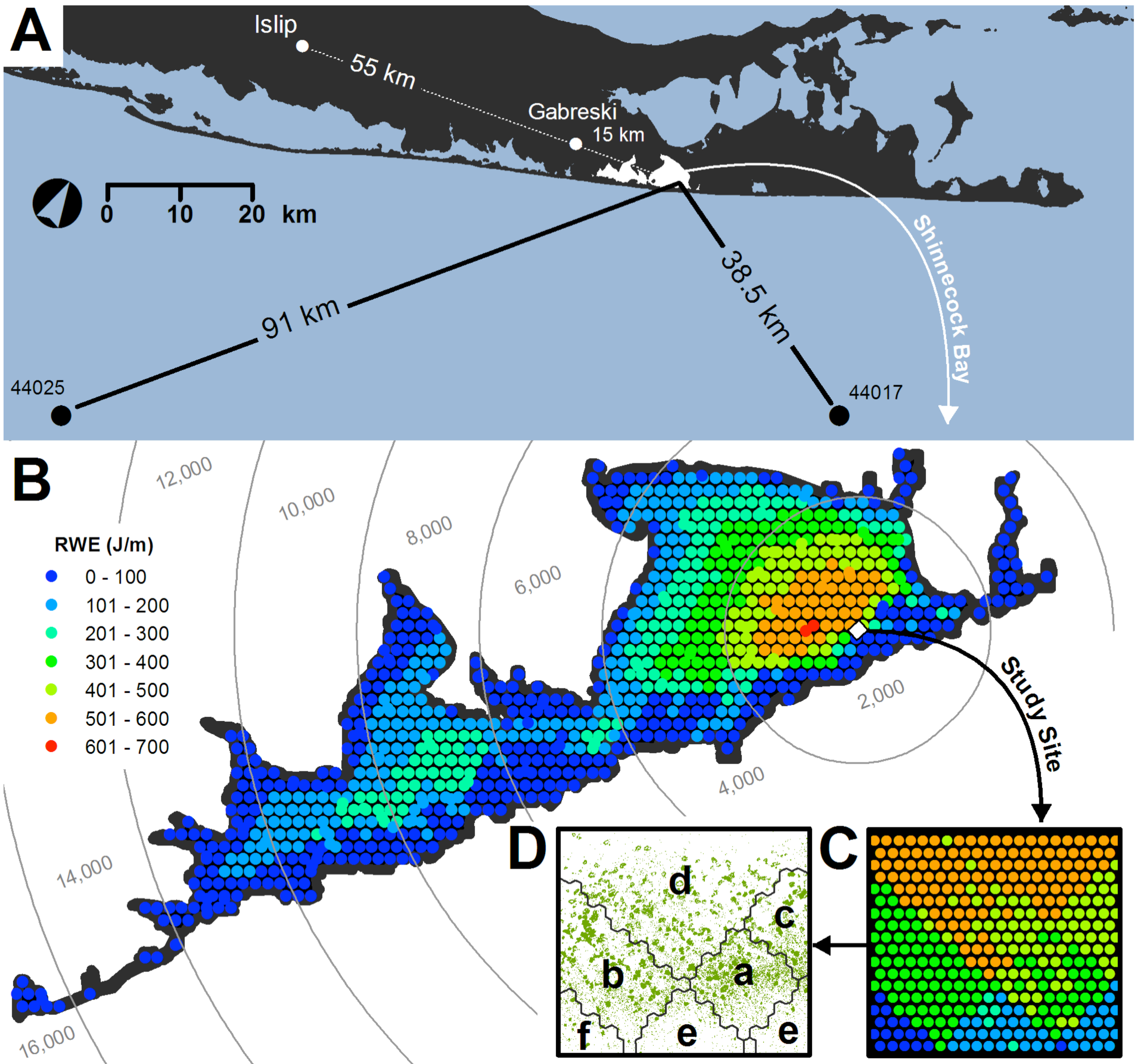

WEMo

200-m grid of 964 positions

12-m grid of 369 positions

Mean Relative Wave Energy (RWE) 2000 to 2014

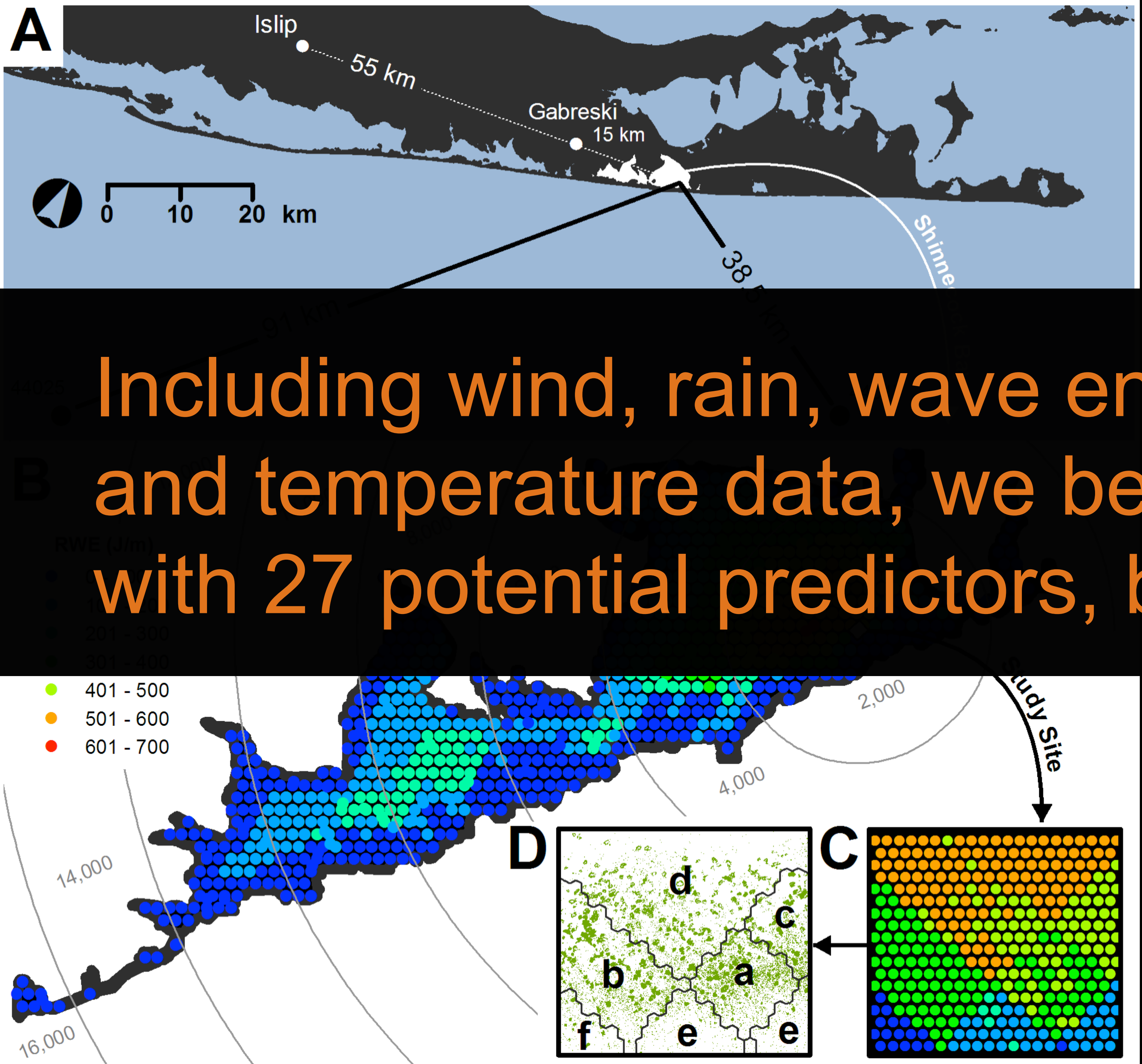

WEMo

200-m grid of 964 positions

Including wind, rain, wave energy and temperature data, we began with 27 potential predictors, but…

Life-History Timeline

Letter size = mean RWE

Letter color = optimal temperature, darker = better

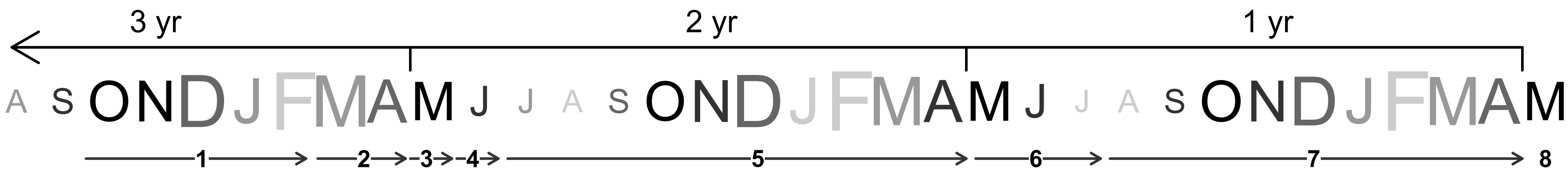

1. Floral induction
2. Flower development
3. Bloom
4. Embryo development and seed release
5. Seed bank
6. Seedling emergence
7. Patch development
8. Patch observed

…when and for how long are they important?

Time-Lagged Predictors

1-month offsets:

up to 1 y

May Jun Jul Aug Sep Oct Nov Dec Jan Feb Mar Apr May

Expanding offsets:

up to 1 y

mean

May Jun Jul Aug Sep Oct Nov Dec Jan Feb Mar Apr May

Yearly offsets:

2 & 3 y

mean

May Jun Jul Aug Sep Oct Nov Dec Jan Feb Mar Apr May

3-month running mean:

up to 3 y

mean

May Jun Jul Aug Sep Oct Nov Dec Jan Feb Mar Apr May

Model Selection

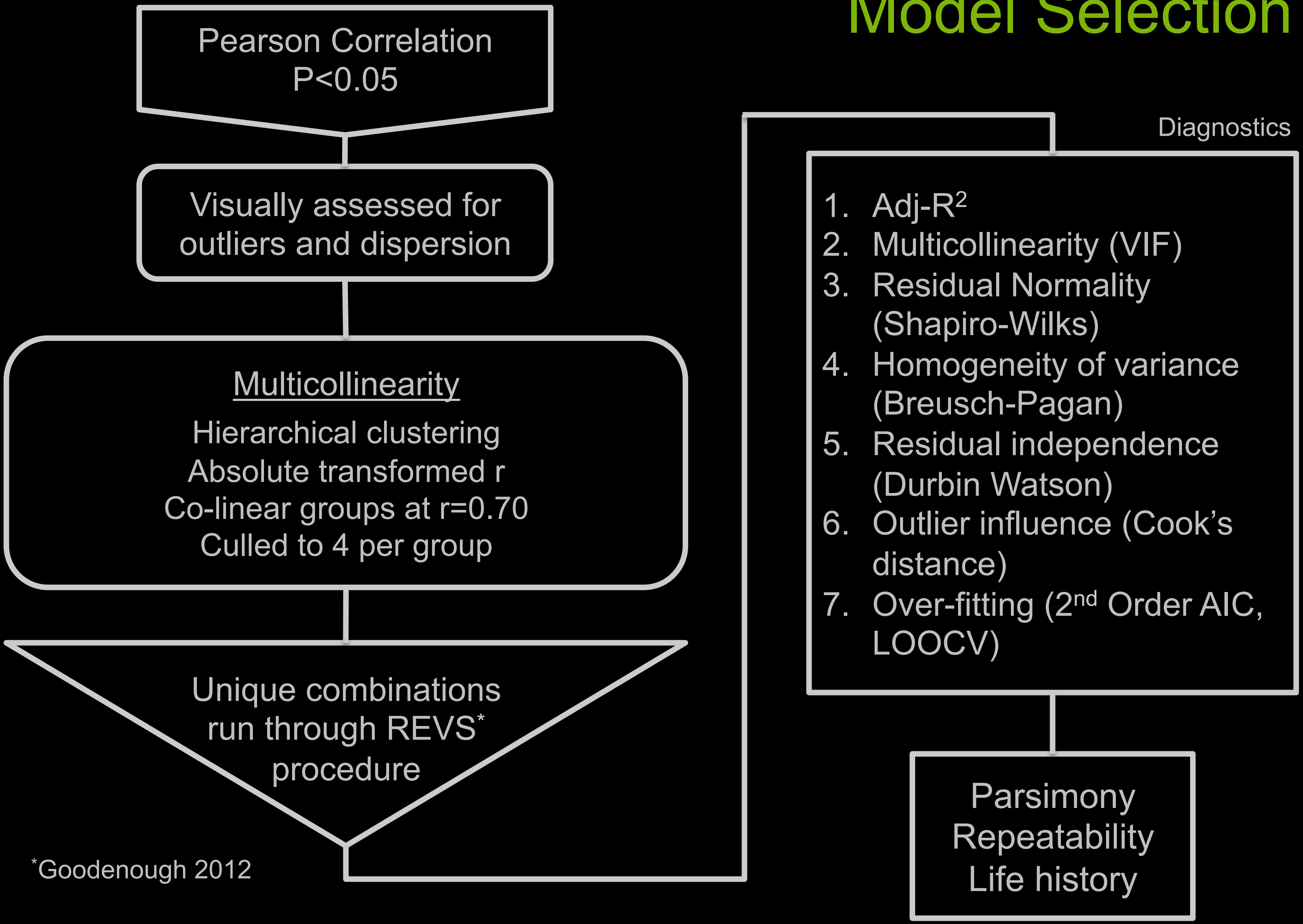

Best-Fit Models

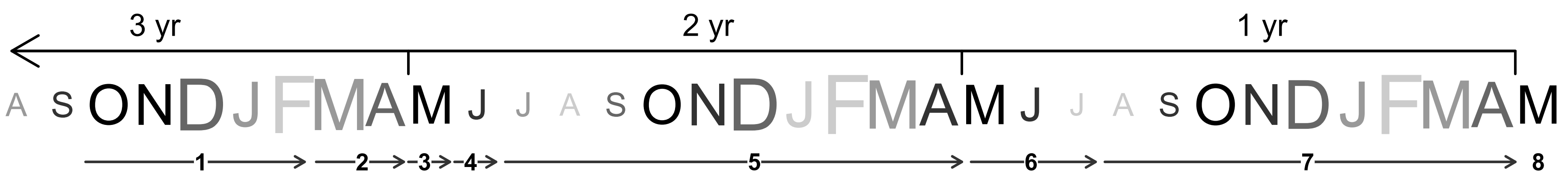

Naked Seed Recruitment (0-6 m):

1. Cold stress (<10° C) during patch development (Sep to Nov) (–)
2. Rainfall during seed bank (Nov to Jan) (+)
3. Easterly wind during previous 3 years (+)

adj-R^2 = 0.998, $P < 0.001$

Rafted Seed Recruitment (>6 m):

1. Wave energy in the 14,000-16,000 m distance band (Nov to April) (–)
2. Wind speed during previous May (–)
3. Wave energy in the 0-2,000-m distance band (previous Feb) (–)

adj-R^2 = 0.981, $P = 0.002$

(Relationship with recruitment)

Comparison

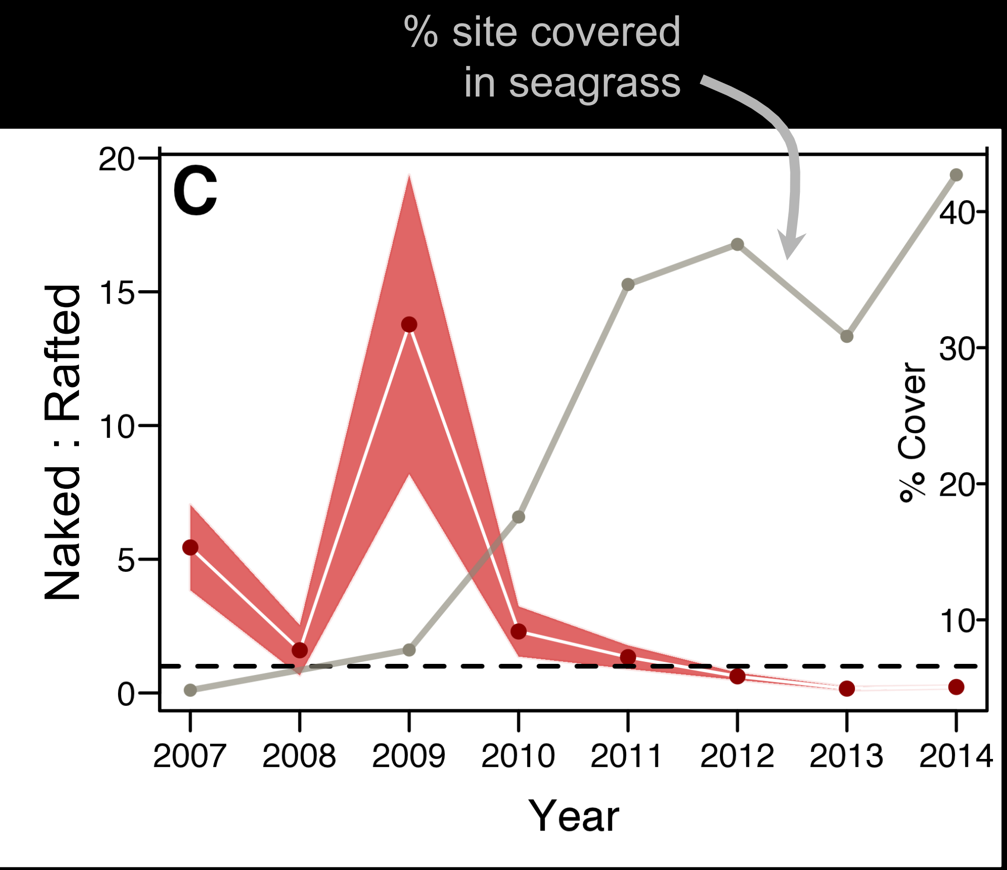

Naked : Rafted

Fixed seagass coverage (10,000 m^2)

Fixed amount of space (20,000 m^2)

Naked > Rafted : 2007 – 2011

Naked < Rafted : 2012 – 2014

Reversal coincided with coverage

1. Rafted dispersal could be limited beyond 4,000 m
2. Artifact of reduced distance space as site filled in
